# Food Intake Changes and Their Impact on Quality of Life in Spanish Citizens with and without COVID-19 during Lockdown

**DOI:** 10.3390/healthcare10081414

**Published:** 2022-07-28

**Authors:** María García-de-Miguel, Elisabet Huertas-Hoyas, Jorge Pérez-Corrales, Cristina Rodríguez-Rivas, Cristina García-Bravo, Sara García-Bravo, Lucía Rocío Camacho-Montaño

**Affiliations:** 1 Centro Neurorrehabilitación Grupo 5, C/Dr. Julián Sanz Ibáñez 57, 50017 Zaragoza, Spain; mgm_96@hotmail.com; 2 Department of Physical Therapy, Occupational Therapy, Rehabilitation and Physical Medicine, Research Group in Evaluation and Assessment of Capacity, Functionality and Disability of Universidad Rey Juan Carlos (TO+IDI), Avenida Atenas s/n, 28922 Alcorcón, Spain; elisabet.huertas@urjc.es (E.H.-H.); cristina.bravo@urjc.es (C.G.-B.); lucia.camacho@urjc.es (L.R.C.-M.); 3 Department of Physical Therapy, Occupational Therapy, Rehabilitation and Physical Medicine, Research Group of Humanities and Qualitative Research in Health Science of Universidad Rey Juan Carlos (Hum&QRinHS), Avenida Atenas s/n, 28922 Alcorcón, Spain; 4 Hospital Fundación Instituto San José, Avenida de la Hospitalidad, s/n, 28054 Madrid, Spain; c.rodriguezr.to@gmail.com; 5 Clínica Physiocare Madrid, Calle José Anespere, 7, 28026 Madrid, Spain; saragbravo@gmail.com

**Keywords:** lockdown, COVID-19, lifestyle, food habits, quality of life, survey

## Abstract

The pervasive impact of the COVID-19 pandemic is just beginning to be analyzed. To date, only a handful of these studies have investigated the relationship between an individual’s quality of life (QoL) and their changes in food intake due to the virus (from the associated symptomatology of diagnosis to the universal impact of lockdown on individual lives, regardless of a person’s health status). Therefore, the purpose of this study is to identify changes in food intake resulting from the pandemic and the corresponding impact on QoL in the Spanish population. This study focuses its investigation on the 3-month time period within which lockdown was its most restrictive, March–May 2020. Survey questions ask participants to compare their eating habits, general health status, and QoL during these 3 months to times prior to the pandemic. We conducted an online survey amongst the Spanish population over 18 years old. Three surveys were administered: (1) the adult eating behavior questionnaire (EABQ), (2) EuroQoL-5D-5L, and (3) The determinants lifestyle changes during lockdown period (March to May 2020). A total of 86 participants were included, with a mean age of 34 years. In the analysis of QoL, significant differences were found according to age, sex, and the presence of a COVID-19 diagnosis. Likewise, in the analysis of food intake, significant differences were found by age (greater changes in the 18–29-years-old population) (*p*< 0.01) and by sex (women *p* < 0.03), as well as greater changes in those with a diagnosis of COVID-19. Furthermore, both food intake and COVID-19 diagnosis were variables that predicted QoL outcomes. In sum, forced home lockdown can cause changes in food intake, which can predict a lower QoL. It follows that the lockdown situation caused by the worldwide COVID-19 pandemic has affected the food intake and overall QoL of the Spanish population.

## 1. Introduction

The coronavirus disease (COVID-19) was stated as a global pandemic in March 2020 by the World Health Organization (WHO) [1]. This disease is characterized by a series of symptoms including fever, cough, dyspnea, myalgia, arthralgia, headache, and diarrhea. Of particular importance to this study, olfactory (anosmia) and gustatory (ageusia) dysfunctions are also prevalent symptoms in patients with COVID-19, affecting up to 20% of the adult population [2]. Anosmia and ageusia have become prevalent symptoms in patients with COVID-19, affecting up to 20% of the adult population [2,3,4]. In addition, due to the rapid spread of COVID-19, social restrictions were decreed. These emergency mandates often minimized mobility, and many countries even imposed lockdown [5].

The restrictions have led to changes in the various lifestyles of the global population. Fear, stress, feelings of loneliness, and/or the economic impact brought about by this situation may contribute to the deterioration of mental health [5]. Some authors have found a high frequency of symptoms of anxiety, stress, or depression during lockdown [6]. These problems often concur with imbalances in eating behavior [5]. This is evidenced by previous studies, which described how people experienced modifications in eating habits during lockdown [5,7,8,9,10,11,12,13,14,15,16,17]. These authors, in turn, associate the changes with psychological problems [16,18]. Gallé et al. [19] reported an increase in unhealthy habits that may have important health consequences in the long term and should be addressed by public health interventions. The study conducted by Rodríguez-Rivas et al. [20] indicates that the COVID pandemic’s restrictions and measures had a negative impact on occupational balance in Spanish citizens and generated in them a modification of habits and routines.

Other authors have focused their research on functional limitations in the olfactory and/or gustatory area produced by COVID-19 [21,22]. As a result of the disease, patients may have difficulties in the perception of tastes or odors [23], leading to detrimental behaviors such as insufficient or unbalanced food intake [21,22]. Previous studies have shown how these disturbances can trigger a reduction in appetite in up to 56% of patients [24]. It follows that the absence of olfactory and/or gustatory senses can reduce the quality of life (QoL) of those affected. 

WHO defines QoL as an “individual’s perception of their position in life in the context of the culture and value system in which they live and in relation to goals, expectations, standards, and concerns” [25]. This definition differs from the definition of well-being, which refers to a complex conceptualization of mental states that includes hedonic dimensions (both positive and negative feelings such as happiness, anxiety, and stress) and eudaemonic dimensions (such as meaningfulness, purposefulness, and worthiness) [26]. Given this distinction between QoL and well-being, previous authors have attributed the trends towards decreased QoL during the pandemic to the restrictions in daily activities necessitated by the virus [23,24]. 

Decreases in the senses of taste and smell can cause people to be unable to appreciate the flavors of food, which limits the enjoyment of this basic activity of daily life [23]. In addition, the sensory distortion of taste and smell can cause negative changes in eating habits in those who require a specific diet, such as people with diabetes or celiac disease, and can even be related to malnutrition in people with cancer [23]. Lastly, changes in an individual’s sense of taste and smell can impact work activities in those professions that require utilization of these senses, such as cooks, wine makers and nurses [24]. Therefore, such symptomatology carries risks for QoL and well-being, since it impairs many important areas of daily life, from eating to social interactions [27].

There are few studies on the changes in food intake caused by a diagnosis of COVID-19 and its associated symptomatology, nor on the general impact of lockdown restrictions on food intake. Therefore, it is not known to what extent these may affect the QoL of individuals. The aim of this study is to identify changes in food intake and their impact on QoL in a cohort of Spanish citizens during lockdown caused by the COVID-19 pandemic, and to analyze these changes by age, sex, and COVID-19 diagnosis.

## 2. Materials and Methods

### 2.1. Design

A cross-sectional design was applied, following the guidelines of the strengthening the reporting of observational studies in epidemiology (STROBE) checklist [28]. This study was approved by the Rey Juan Carlos University Research Ethics Committee, reference number: 1301202101721. All study participants were informed verbally and in writing by means of informed consent about the purpose of the study and the methodology of data collection and analysis. In addition, they were informed about the processing of personal data referring to the current data protection law, being able to revoke their consent to participate in the study at any time. The signing of the informed consent by the participants before starting the study was carried out following the guidelines of the revision of the Declaration of Helsinki, carried out by the World Medical Association Assembly in 2013 [29].

### 2.2. Study Sample

Participants were recruited through consecutive- and snowball-sampling approaches over a period of one month (19 February–8 March 2021) through social media (Facebook, WhatsApp and Instagram). The inclusion criteria for participants were (1) to be over 18 years of age; (2) to have resided in Spain during the period of lockdown between March–May 2020; (3) to have signed the informed consent form. Exclusion criteria were: (1) presenting subjective amnesic or attentional deficits; and (2) the presence of other diseases (apart from COVID-19) that prevented participation in activities of daily living during the last 5 months. 

The sample was further divided into two groups: COVID-19 and non-COVID-19 (the healthy sample served as the control). The COVID-19 sample was composed of people with a prior diagnosis of COVID-19. A specific exclusion criterion of this sample was suffering from ageusia and/or anosmia due to a diagnosis other than COVID-19.

### 2.3. Procedure

Data were collected through an online survey created with the Microsoft Forms application from Office365 (Microsoft Office 365). Sociodemographic data were collected, as well as data on participants’ eating habits, general health status, and quality of life (QoL). The questions referred to the period before the COVID-19 pandemic and to the period of lockdown from March to May 2020.

### 2.4. Instruments

The adult eating behavior questionnaire (AEBQ): a questionnaire that assesses the eating behavior of adults. The individual assigns each item a score according to his or her perception, with the ranking as follows: (1) they strongly disagree, (2) they disagree, (3) they neither agree nor disagree, (4) they agree, or (5) they completely agree. Questions are worded in such a way that the higher the final score obtained, the better the eating behavior of the participant [30]. This outcome measure has a valid and reliable psychometric questionnaire for measuring appetitive traits in a Mexican Spanish-speaking population (Supplementary Material Table S1) [30].

Determinants of the lifestyle changes during COVID-19: a questionnaire that assesses changes in activities of daily living, including eating, due to the COVID-19 pandemic. It is a questionnaire through which information is obtained on the food intake changes due to COVID-19 and the resultant lockdown [31].

The EuroQol 5D-5L instrument (EQ-5D-5L): The EQ-5D-5L questionnaire assesses QoL, both during the pre-pandemic period and during the pandemic. It is comprised of five dimensions (mobility, self-care, activities of daily living, pain and/or discomfort, and anxiety and/or depression) scored on five levels. The values range from 1 to 5, distributed in the following order: 1, “no problems”; 2, “mild problems”; 3, “moderate problems”; 4, “severe problems”; and 5, “extreme problems.” The higher the score, the worse the quality of life (QoL). The total score is obtained using the tool’s own software [32] (Supplementary Material Table S2).

### 2.5. Data Analysis

Descriptive data were reported by the qualitative variables and the mean and standard deviation of the continuous variables. Non-normal distribution was confirmed by the Shapiro–Wilk test. Differences between the pre-lockdown period and during lockdown were analyzed using the Wilcoxon test for related samples on quantitative variables in each group (COVID-19 diagnosis and non-COVID-19). Effect size was calculated using Cohen’s d. Both non-parametric and non-related samples methods were used to study the differences between variables (sex/COVID-19 diagnosis/age/regions of Spain/level of physical activity) using the Mann–Whitney U or Kruskal–Wallis test to compare these data between all participants. Chi-square tests were conducted to compare the changes in the proportion of COVID-19 and non-COVID-19 in relation to quantity of eating, quality of nutrition, and physical activity. Spearman’s statistic was used to examine the correlations between variables. For the multiple regression model, the R, R^2^, R^2^ adjusted, F-test, regression coefficient (b), standardized beta (std. b), and t-test were reported. Durbin–Watson tests helped to analyze the residuals for establishing the robustness among the tested models. Significance was determined with a 2-tailed *p*-value of 0.05. The analysis of the study results was carried out using the statistical program IBM SPSS Statistics for Windows, version 27.0 (Copyright © 2022 IBM SPSS Corp.).

## 3. Results

There were 86 participants, mostly from Aragón (46.9%), located in northern Spain, with a mean age of 34 years (34.32 ± 14.305). The sociodemographic data are shown in Table 1.

Differences between the pre-lockdown period and lockdown period were analyzed. According to the results, significant differences between periods (*p* < 0.000) were found in almost all variables. AEBQ outcomes during confinement were significantly worse than before in the COVID-19 sample but not in non-COVID-19 sample (Table 2). Moreover, there were differences between the two groups COVID-19 and non-COVID-19 sample during confinement but not in pre-pandemic period (Supplementary Material Table S3). QoL outcomes during confinement more severely deteriorated in the COVID-19 sample (Table 2).

Regarding quantified changes in food intake, only 37% of the COVID-19 sample reported having eaten the same amount as usual, compared to 60% of the healthy sample. Specifically, the COVID-19 sample ate less than usual during lockdown. When comparing the quality of nutrition of both samples, 18.8% of those who had the disease rated their nutrition as worse, and 56% stated that they had skipped meals because they were not hungry during the disease. In COVID-19 sample, 16% stated that they did not eat regular meals during lockdown compared to 2% of the non-COVID-19 sample. 

**Figure 1:**
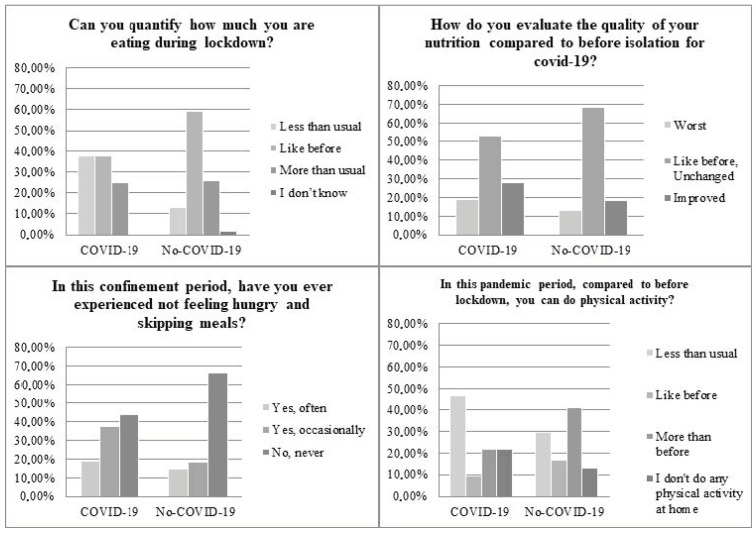
Determinants of the lifestyle changes during COVID-19. Note: A Chi-Square Test was performed to determine whether the proportions were equal between COVID-19 and Non-COVID-19. The proportions only differed significantly in the quantity of eating, X^2^ (3, n = 60) = 7934, *p* = 0.047.

In addition, 47% of the COVID-19 sample reported having performed less exercise than usual, and 22% did not do any physical exercise. In contrast, 13% of the non-COVID-19 sample rated their nutrition as worse, and 67% of them never skipped meals during lockdown. In addition, in the non-COVID-19 sample, only 13% did not exercise, and 30% reported to have exercised less (Figure 1). In the COVID-19 sample, 91% bought fresh food and vegetables more frequently before confinement while 50% did during confinement. In the non-COVID-19 sample, 93% bought fresh food and vegetables more frequently before confinement while 65% did during confinement. A total of 20% of the COVID-19 sample purchased more cakes, biscuits, and sweet or savory snacks during confinement compared to 2% pre-confinement, whereas it was 15% of the non-COVID-19 sample during lockdown compared to 2% pre-confinement. The decrease in the consumption of fresh foods and vegetables and the increased consumption of ultra-processed foods rich in sugars, trans fats and salt show a worsening in the nutritional quality of the diet.

Results of the analysis of AEBQ scores and different demographic variables (sex, COVID-19, age, place of residence, and physical activity) can be found in Table S3 (Supplementary Material Table S3). With respect to sex, the differences before lockdown were significant. However, during lockdown, these differences were not found again. In contrast, for COVID-19 diagnosis, there were no differences between groups before the pandemic, whereas during lockdown, people who had been diagnosed with COVID-19 had significantly lower scores. Regarding eating habits with respect to age range, the group of individuals 36 years old and older scored significantly worse during lockdown than the group between 18 and 29 years old. Although not significant, variations in food intake scores were found with respect to the region of residence. Before the pandemic, scores in Northern and Central Spain were similar, while in the south they were higher. However, these differences were not significant during lockdown; the northern zone decreased only slightly, while the central and southern zones decreased considerably, with the central zone being the most affected. With respect to physical activity, the food intake scores before the pandemic and scores during lockdown were similar. However, those who did not engage in any physical activity during lockdown decreased by 10 points on the AEBQ compared to those who reported the same activity as before the pandemic (Supplementary Material Table S3). When testing for correlations between the AEBQ and QoL (according to the EQ-5D-5L), the data showed a significant positive relationship during the lockdown only in the COVID-19 group (Table 3).

A simple regression model was carried out to examine the prediction of AEBQ score in the COVID-19 and non-COVID-19 groups on the outcomes of QoL. The EQ-5D-5L presented a significant relationship with AEBQ score as a predictor only in the COVID-19 group. R^2^ accounts for 25% of the variance in the COVID-19 regression model (Table 4).

## 4. Discussion

The aim of the present study was to identify changes in food intake and their impact on QoL in Spanish citizens with and without COVID-19 during the COVID-19 lockdown, and to analyze these changes by age, sex, and the presence of COVID-19 diagnosis. The results show decreased food intake and decreased QoL during lockdown in both COVID-19 and non-COVID-19 samples, with the COVID-19 sample experiencing a greater decrease in both categories. The data also show the COVID-19 group presenting significantly worse eating-habit scores than the non-COVID-19 group during lockdown. This suggests that food intake is more affected by a COVID-19 diagnosis than by simply living in lockdown.

Previous studies described the olfactory and gustatory dysfunctions that can be generated by COVID-19 [3,21,22,23]. In this regard, possible difficulties in appreciating both the taste and smell of food have been described. This loss may make it more difficult for people to enjoy food, and they may even avoid it, thus altering their eating habits [23]. This is due to a decrease in appetite related to the loss of pleasure during eating [23,33], and it may also produce a faster feeling of satiety related to the decrease in the palatability of food [34]. However, the study by Høier et al. [34] shows how although this is the general trend, there are also people who experience an increase in appetite that causes an increase in food intake.

Our results indicate that a high percentage of the COVID-19 group underwent a change in food intake. More than half of the COVID-19 group claimed to have skipped meals because they were not hungry during the illness. In congruence with Chaaban’s study developed in Denmark, the patients themselves associated this with a lack of appetite, along with other symptoms such as nausea and shortness of breath as possible causes [33]. In our study, a lower proportion than the COVID-19 sample, 33% of the healthy sample, reported having skipped meals. These results are consistent with previous studies [35,36] indicating that COVID-19 infection is associated with a change in eating habits, which could also be related to emotional aspects such as anxiety [37].

However, other authors observed an increase in the number of main meals [15]. Regarding this minority of the COVID-19 sample who reported eating more than before, the cause could be due to the need to find flavors that fulfill their sensory desires, as also indicated by Chaaban in his study [33]. These results agree with the study by Saals et al. [38] in which, although the regularity in meals decreased, the frequency, especially of the consumption of snacks, increased. In the non-COVID-19 group, only a small percentage of individuals presented changes between the amount of food they consumed before the pandemic in comparison to lockdown. However, within this small percentage, a significant difference was seen. Of this minority, a majority (26%) experienced an increase in consumption, as indicated by other studies investigating the period of lockdown [13,14,32,33]. Other authors [12,39] found that most improved the quality of their nutrition during lockdown. Although 13% of the non-COVID-19 group claimed to have worsened the quality of their diet during lockdown, 20% claimed an improvement, and reported better adherence to a Mediterranean diet during lockdown [8,11,16]. Despite these inconsistencies, both aspects could be related to the lack of habits and routines during lockdown [10,12]. These differences, however, were not significant.

As for the findings regarding physical activity during lockdown, we also observed a wide percentage difference between those respondents who had been infected with COVID-19 and those who had only been through the lockdown decreed by the Spanish authorities. Nevertheless, the latter also performed less exercise than usual, in accordance with other studies that investigated the period of lockdown [8,16,19]. Before the pandemic, the level of physical exercise practiced per week did not influence food intake scores. However, even though the differences were not significant, we found that during lockdown, those who did not perform any type of exercise were also those with worse food intake. The insignificant differences between samples may be due to the insufficient group sizes, since the differences in the scores are considerable, as represented by the data.

The QoL of our sample significantly deteriorated in both groups; however, it was more significant in people who had suffered COVID-19, with a 43% decrease in the percentage of their overall QoL. This coincides with other studies that analyzed the QoL in people who had been diagnosed with COVID-19 [40,41,42], which also showed that those infected with COVID-19 experienced decreased QoL in comparison to their non-COVID-19 counterparts [40]. However, these scores were slightly lower than ours [41]. In our study, the results of analyzing the group as a whole show a significant decrease in QoL despite being lower than the COVID-19 sample, thus correlating with other studies [43,44,45], although the level of QoL determined by the EuroQoL in these studies was higher than in our sample.

Regarding the analysis of the data according to sex, we observed that males had significantly worse food intake than females before the pandemic. During lockdown, these differences were no longer significant, since, although males had a slight decrease in the score, females worsened in this behavior. The results for the period of lockdown are similar to the study by Sidor et al. [14], which investigated changes in food consumption for a cohort of Polish citizens, in which no differences were found according to sex. Before the pandemic, there were no differences in the sample of people who were subsequently diagnosed with COVID-19 in comparison to the healthy sample. However, during lockdown, the food intake of people who had been diagnosed with COVID-19 began to experience a greater deterioration than that of the healthy group, suggesting that having COVID-19 has consequences for eating.

Prior to lockdown, there were no significant differences between food intake between age groups. However, during lockdown, the older group (30–68 y.o.) experienced a greater decrease in food intake than the younger group (18–29 y.o.). These findings are in line with similar studies by Di Renzo et al. [35] and Sidor et al. [14]. However, they differ from the study by Rodriguez-Perez et al. [16] on the Spanish population, wherein the elderly participants maintained greater adherence to the Mediterranean diet than younger participants throughout the lockdown period.

This is one of the few studies to focus on the impact of the COVID-19 pandemic on food intake and its repercussions on QoL. The only study we have found that focuses on the impact of COVID-19 on food intake is limited to comparing eating habits of healthy individuals to those of people diagnosed with COVID-19 [31]. Our study goes beyond this, and finds the greater the imbalance in food intake, the worse the QoL outcomes. Even more, this research indicates that food intake could predict an individual’s QoL scores. We detected possible factors related to worse eating during the pandemic, such as diagnosis of COVID-19, old age, and lack of physical activity. Little evidence has been identified in this area, so further studies are required to corroborate the results obtained in the present work with a larger sample size. However, this can only be achieved in future situations of social isolation. While this study has shown a significant association between food intake and QoL, further studies on the impact of COVID-19 on eating and QoL are needed to provide a basis for the treatment of eating habits, and in turn, for the improvement in QoL.

As practical recommendations based on our findings, we consider it essential to carry out daily and weekly meal planning, preferably guided by a nutrition and dietetic professional. However, we are aware that this option is not always possible, so as general recommendations, in line with the specific recommendations of international organizations [46], we recommend adequate hydration (1.5–2 L/day), consumption of fruit and vegetables at least five times a day, building a colorful plate that stimulates the view to the detriment of the possible affectation of taste and smell, and the choice of whole grains instead of refined cereals. In relation to protein foods, the consumption of legumes, fish and white meat is preferable to red meat and preserves. Additionally, of course, healthy fats such as nuts or extra virgin olive oil should be chosen and ultra-processed foods rich in sugars, trans fats and salt should be limited. In order for this healthy consumption of food to be fulfilled, it would be necessary to emphasize the importance of decision making during the purchase of food, with strategies such as preparing a shopping list beforehand, making purchases at times when the person does not feel hungry and/or anxious or make purchases in greengrocers, fishmongers or butchers in local markets instead of in large supermarkets with a high presence of ultra-processed foods. During lockdown, it was not always possible to shop in a market, so a planned purchase of healthy food online is an option to consider. Finally, at a structural level, it would be necessary for government institutions to implement a greater number of awareness campaigns on healthy eating that contribute to improving the diet of society.

## 5. Limitations

When discussing the findings of this study, the limitations must be considered. First, in relation to sample selection bias, it was found more difficult to access older adults than young people and adults. In cross-sectional studies, a non-representative sample is a frequent cause of selection bias. Nevertheless, we attempted to avoid this bias by clearly identifying the study populations and preventing the number of non-responses [47]. Second, the sample size was small, with little geographic variety, so there may not be enough of a population to justify generalization of the results. Third, due to the mobility restrictions mandated throughout the period of data collection, we implemented an online survey which is a widely used methodology during the pandemic period [35,44]. However, this method could have given rise to potential bias, as it relies on self-report rather than objective measures. Even more, data about the pre-pandemic period were elicited by retrospective questions which can lead to retrospective or memory bias. This limitation is seen across studies of the COVID-19 pandemic. Fourth, the snowball-sampling approach can lead to bias, although consecutive sampling could reduce volunteerism and other selection biases [48].

Moreover, a tool that has not yet been validated was applied to assess changes in lifestyle-related behavior during the COVID-19 lockdown [31], despite it being used as a complementary survey to the other tools to collect qualitative information during confinement.

Despite these limitations, this study also shows many strengths. To our knowledge, this is the first study reporting a first analysis of the importance of food intake in people with COVID-19 compared to those without. We were able to mitigate the social desirability bias by making the survey anonymous. This online methodology allowed us to study how social isolation affects changes in the dietary intake of people in confinement. Furthermore, this study can contribute to improving the design of interventions in diseases which also require periods of social isolation.

It would be necessary to carry out a greater number of longitudinal studies that detect the effects of confinement on the habits and quality of life of the population. However, due to the exceptional nature of the isolation situation caused by the pandemic at a specific historical moment, it is possible that these studies cannot be repeated in the short term.

## 6. Conclusions

Our results reveal that food intake has been affected more in individuals with a diagnosis of COVID-19 than in those who only had to live in lockdown. Similarly, QoL more severely deteriorated in the COVID-19 sample, and was significantly related to changes in individuals’ food intake and eating behaviors. Alterations in food intake, in turn, significantly predicted a lower QoL. Therefore, based on these findings, addressing food intake can contribute to a better QoL. Lastly, this study shows how not only a previous diagnosis of COVID-19, but also an individual’s age and level of physical activity, can influence food intake and behavior, providing information to be considered in the development of specialized intervention programs.

## Figures and Tables

**Table 1:** Socio-demographic characteristics of the sample (*n* = 86).

Characteristics	Complete Sample (*n* = 86)	COVID-19 Sample (*n* = 32)	Non-COVID-19 Sample (*n* = 54)
Age	34.32 ± 14.305	34.42 ± 12.096	34.26 ± 15.563
Age range			
18–29 years		20 (62.5%)	30 (55.6%)
30–68 years		11 (34.4%)	23 (42.6%)
Missing data		1 (3.1%)	1 (1.9%)
Sex			
Women	67 (77.9%)	23 (71.9%)	44 (81.5%)
Men	19 (22.1%)	9 (28.1%)	10 (18.5%)
Place of residence			
Aragón	35 (40.7%)	15 (46.9%)	20 (37%)
Madrid	28 (32.6%)	9 (28.1%)	19 (35.2%)
Other	23 (26.7%)	8 (25%)	15 (27.8%)
Marital Status			
Single	22 (22.9%)	9 (28.1%)	13 (24.1%)
Couple	37 (38.5%)	11 (34.4%)	26 (48.1%)
Married	24 (25%)	10 (31.3%)	14 (25.9%)
Separated	2 (2.1%)	2 (6.3%)	
Divorced	1 (1%)		1 (1.9%)

Note: % = percentage of persons. Other: Andalucia, Castilla y León, Castilla-La Mancha, Islas Canarias, Navarra, Valencia, Cataluña, Murcia, País Vasco, La Rioja.

**Table 2:** Analysis of differences in the sample pre-pandemic period and during lockdown (n = 86).

	COVID-19 Sample n = 32				Non-COVID-19 Sample n = 54			
	M ± SD	Z	*p*	*d*	M ± SD	Z	*p*	*d*
AEBQ		−3.581	0.000	0.63		−0.426	0.670	0.06
Before lockdown	64.29 ± 7.506				64.96 ± 7.256			
Home confinement	57.06 ± 5.332				64.70 ± 8.132			
EuroQoL		−4.258	0.000	0.75		−5.441	0.000	0.74
Before lockdown	0.91 ± 0.129				0.925 ± 0.107			
Home confinement	0.49 ± 0.278				0.743 ± 0.172			

Note: M = median, SD = standard deviation, Z = statistical value, *p* = value of significance, d = Cohen’s d.

**Table 3:** Correlations between AEBQ and EuroQoL scores during lockdown.

	COVID-19	Non-COVID-19
EuroQoL/ AEBQ home confinement	**0.440** *	−0.141

Note: Spearman’s ρ; denotes significant correlations at the 0.01 level (2-tailed); * denotes significant correlations at the 0.05 level (2-tailed). Bold values are statistically significant.

**Table 4:** Results from multiple regression examining the AEBQ score as predictor of EuroQoL 5D-5L outcomes during lockdown.

Model	R	R^2^	R^2^ Adjusted	Standard Error of Estimation	F-Test	*p*	Durbin–Watson	Predictor	b (95% CI)	Std. b	*t*-Test	*p*
COVID-19	0.498	0.248	0.216	0.242	7597	0.011	2065	(Constant) AEBQ	−0.814 (−1796, 0.168) 0.023 (0.006, 0.039)	0.498	−1715 2756	0.011 0.100
NonCOVID-19	0.088	0.008	−0.012	0.181	0.393	0.534	2316	(Constant) AEBQ	0.867 (0.441, 1293) −0.002 (−0.009, 0.005)	−0.088	4085 −0.627	0 0.534

Note: R = correlation coefficient, R^2^ = determination coefficient, F-test = statistical value, Durbin–Watson = test statistic, b = Beta standardized coefficient, Std. b = standard deviation, t-test = statistical value, *p* = value of significance.

## Data Availability

Data are available upon reasonable request to the corresponding author.

## Supplementary Materials

The following supporting information can be downloaded at: https://www.mdpi.com/article/10.3390/healthcare10081414/s1, Table S1: Adult Eating Behavior Questionnaire; Table S2: The EuroQol 5D-5L instrument; Table S3: Analysis of differences between independent samples in the AEBQ.
