# Supplementary material for: Food Intake Changes and Their Impact on Quality of Life in Spanish Citizens with and without COVID-19 during Lockdown
Source: Healthcare (Basel). 2022 Jul 28;10(8):1414. doi: 10.3390/healthcare10081414 (PMC9408131; doi:10.3390/healthcare10081414)
Supplement: Supplementary file 1 [file healthcare-10-01414-s001.zip › Supplementary material Table S1.pdf]

Adult Eating Behaviour Questionnaire

Please read each statement and tick the box most appropriate to you

	Strongly disagree	Disagree	Neither agree or disagree	Agree	Strongly agree
I love food	☐	☐	☐	☐	☐
I often decide that I don't like a food, before tasting it	☐	☐	☐	☐	☐
I enjoy eating	☐	☐	☐	☐	☐
I look forward to mealtimes	☐	☐	☐	☐	☐
I eat more when I'm annoyed	☐	☐	☐	☐	☐
I often notice my stomach rumbling	☐	☐	☐	☐	☐
I refuse new foods at first	☐	☐	☐	☐	☐
I eat more when I'm worried	☐	☐	☐	☐	☐
If I miss a meal I get irritable	☐	☐	☐	☐	☐
I eat more when I'm upset	☐	☐	☐	☐	☐
I often leave food on my plate at the end of a meal	☐	☐	☐	☐	☐
I enjoy tasting new foods	☐	☐	☐	☐	☐
I often feel hungry when I am with someone who is eating	☐	☐	☐	☐	☐
I often finish my meals quickly	☐	☐	☐	☐	☐
I eat less when I'm worried	☐	☐	☐	☐	☐
I eat more when I´m anxious	☐	☐	☐	☐	☐
Given the choice, I would eat most of the time	☐	☐	☐	☐	☐

	Strongly disagree	Disagree	Neither agree or disagree	Agree	Strongly agree
I eat less when I'm angry	☐	☐	☐	☐	☐
I am interested in tasting new food I haven't tasted before	☐	☐	☐	☐	☐
I eat less when I'm upset	☐	☐	☐	☐	☐
I eat more when I'm angry	☐	☐	☐	☐	☐
I am always thinking about food	☐	☐	☐	☐	☐
I often get full before my meal is finished	☐	☐	☐	☐	☐
I enjoy a wide variety of foods	☐	☐	☐	☐	☐
I am often last at finishing a meal	☐	☐	☐	☐	☐
I eat more and more slowly during the course of a meal	☐	☐	☐	☐	☐
I eat less when I'm annoyed	☐	☐	☐	☐	☐
I often feel so hungry that I have to eat something right away	☐	☐	☐	☐	☐
I eat slowly	☐	☐	☐	☐	☐
I cannot eat a meal if I have had a snack just before	☐	☐	☐	☐	☐
I get full up easily	☐	☐	☐	☐	☐
I often feel hungry	☐	☐	☐	☐	☐
When I see or smell food that I like, it makes me want to eat	☐	☐	☐	☐	☐
If my meals are delayed I get light-headed	☐	☐	☐	☐	☐
I eat less when I'm anxious	☐	☐	☐	☐	☐

Adult Eating Behaviour Questionnaire - Scoring information

		Strongly disagree	Disagree	Neither agree or disagree	Agree	Strongly agree
EF	I love food	☐	☐	☐	☐	☐
FF	I often decide that I don’t like a food, before tasting it	☐	☐	☐	☐	☐
EF	I enjoy eating	☐	☐	☐	☐	☐
EF	I look forward to mealtimes	☐	☐	☐	☐	☐
EOE	I eat more when I'm annoyed	☐	☐	☐	☐	☐
H	I often notice my stomach rumbling	☐	☐	☐	☐	☐
FF	I refuse new foods at first	☐	☐	☐	☐	☐
EOE	I eat more when I'm worried	☐	☐	☐	☐	☐
H	If I miss a meal I get irritable	☐	☐	☐	☐	☐
EOE	I eat more when I'm upset	☐	☐	☐	☐	☐
SR	I often leave food on my plate at the end of a meal	☐	☐	☐	☐	☐
FF*	I enjoy tasting new foods	☐	☐	☐	☐	☐
FR	I often feel hungry when I am with someone who is eating	☐	☐	☐	☐	☐
SE*	I often finish my meals quickly	☐	☐	☐	☐	☐
EUE	I eat less when I'm worried	☐	☐	☐	☐	☐
EOE	I eat more when I´m anxious	☐	☐	☐	☐	☐
FR	Given the choice, I would eat most of the time	☐	☐	☐	☐	☐
EUE	I eat less when I'm angry	☐	☐	☐	☐	☐
FF*	I am interested in tasting new food I haven’t tasted before	☐	☐	☐	☐	☐
EUE	I eat less when I'm upset	☐	☐	☐	☐	☐
EOE	I eat more when I'm angry	☐	☐	☐	☐	☐
FR	I am always thinking about food	☐	☐	☐	☐	☐
SR	I often get full before my meal is finished	☐	☐	☐	☐	☐
FF*	I enjoy a wide variety of foods	☐	☐	☐	☐	☐
SE	I am often last at finishing a meal	☐	☐	☐	☐	☐
SE	I eat more and more slowly during the course of a meal	☐	☐	☐	☐	☐

		Strongly disagree	Disagree	Neither agree or disagree	Agree	Strongly agree
EUE	I eat less when I'm annoyed	□	□	□	□	□
H	I often feel so hungry that I have to eat something right away	□	□	□	□	□
SE	I eat slowly	□	□	□	□	□
SR	I cannot eat a meal if I have had a snack just before	□	□	□	□	□
SR	I get full up easily	□	□	□	□	□
H	I often feel hungry	□	□	□	□	□
FR	When I see or smell food that I like, it makes me want to eat	□	□	□	□	□
H	If my meals are delayed I get light-headed	□	□	□	□	□
EUE	I eat less when I'm anxious	□	□	□	□	□

* Reversed items

Enjoyment of food	=	item mean EF
Emotional over-eating	=	item mean EOE
Emotional under-eating	=	item mean EUE
Food fussiness	=	item mean FF
Food responsiveness	=	item mean FR
Slowness in eating	=	item mean SE
Hunger	=	item mean H
Satiety responsiveness	=	item mean SR

Strongly disagree =1, Disagree = 2,Neither agree nor disagree = 3, Agree =4, Strongly agree =5

Adult Eating Behaviour Questionnaire - Scoring information

		Strongly disagree	Disagree	Neither agree or disagree	Agree	Strongly agree
EF	I love food	◻	◻	◻	◻	◻
EF	I enjoy eating	◻	◻	◻	◻	◻
EF	I look forward to mealtimes	◻	◻	◻	◻	◻
EOE	I eat more when I'm annoyed	◻	◻	◻	◻	◻
EOE	I eat more when I'm worried	◻	◻	◻	◻	◻
EOE	I eat more when I'm upset	◻	◻	◻	◻	◻
EOE	I eat more when I´m anxious	◻	◻	◻	◻	◻
EOE	I eat more when I'm angry	◻	◻	◻	◻	◻
EUE	I eat less when I'm worried	◻	◻	◻	◻	◻
EUE	I eat less when I'm angry	◻	◻	◻	◻	◻
EUE	I eat less when I'm upset	◻	◻	◻	◻	◻
EUE	I eat less when I'm annoyed	◻	◻	◻	◻	◻
EUE	I eat less when I'm anxious	◻	◻	◻	◻	◻
FF	I often decide that I don’t like a food, before tasting it	◻	◻	◻	◻	◻
FF	I refuse new foods at first	◻	◻	◻	◻	◻
FF*	I enjoy tasting new foods	◻	◻	◻	◻	◻
FF*	I am interested in tasting new food I haven’t tasted before	◻	◻	◻	◻	◻
FF*	I enjoy a wide variety of foods	◻	◻	◻	◻	◻
FR	I often feel hungry when I am with someone who is eating	◻	◻	◻	◻	◻
FR	Given the choice, I would eat most of the time	◻	◻	◻	◻	◻
FR	I am always thinking about food	◻	◻	◻	◻	◻
FR	When I see or smell food that I like, it makes me want to eat	◻	◻	◻	◻	◻
H	I often notice my stomach rumbling	◻	◻	◻	◻	◻
H	If I miss a meal I get irritable	◻	◻	◻	◻	◻
H	I often feel so hungry that I have to eat something right away	◻	◻	◻	◻	◻
H	I often feel hungry	◻	◻	◻	◻	◻

		Strongly disagree	Disagree	Neither agree or disagree	Agree	Strongly agree
H	If my meals are delayed I get light-headed	☐	☐	☐	☐	☐
SE	I am often last at finishing a meal	☐	☐	☐	☐	☐
SE	I eat more and more slowly during the course of a meal	☐	☐	☐	☐	☐
SE	I eat slowly	☐	☐	☐	☐	☐
SE*	I often finish my meals quickly	☐	☐	☐	☐	☐
SR	I often leave food on my plate at the end of a meal	☐	☐	☐	☐	☐
SR	I often get full before my meal is finished	☐	☐	☐	☐	☐
SR	I cannot eat a meal if I have had a snack just before	☐	☐	☐	☐	☐
SR	I get full up easily	☐	☐	☐	☐	☐
