# Supplementary material for: Food Intake Changes and Their Impact on Quality of Life in Spanish Citizens with and without COVID-19 during Lockdown
Source: Healthcare (Basel). 2022 Jul 28;10(8):1414. doi: 10.3390/healthcare10081414 (PMC9408131; doi:10.3390/healthcare10081414)
Supplement: Supplementary file 1 [file healthcare-10-01414-s001.zip › Supplementary material Table S2.pdf]

Health Questionnaire (EQ-5D-5L)

Under each heading, please tick the ONE box that best describes your health TODAY.

MOBILITY

☐1 I have no problems in walking about

☐2 I have slight problems in walking about

☐3 I have moderate problems in walking about

☐4 I have severe problems in walking about

☐5 I am unable to walk about

SELF-CARE

☐1 I have no problems washing or dressing myself

☐2 I have slight problems washing or dressing myself

☐3 I have moderate problems washing or dressing myself

☐4 I have severe problems washing or dressing myself

☐5 I am unable to wash or dress myself

USUAL ACTIVITIES *(e.g. work, study, housework, family or leisure activities)*

☐1 I have no problems doing my usual activities

☐2 I have slight problems doing my usual activities

☐3 I have moderate problems doing my usual activities

☐4 I have severe problems doing my usual activities

☐5 I am unable to do my usual activities

PAIN / DISCOMFORT

☐1 I have no pain or discomfort

☐2 I have slight pain or discomfort

☐3 I have moderate pain or discomfort

☐4 I have severe pain or discomfort

☐5 I have extreme pain or discomfort

ANXIETY / DEPRESSION

☐1 I am not anxious or depressed

☐2 I am slightly anxious or depressed

☐3 I am moderately anxious or depressed

☐4 I am severely anxious or depressed

☐5 I am extremely anxious or depressed

UK (English) © 2009 EuroQol Group EQ-5D™ is a trade mark of the EuroQol Group

Health Questionnaire (EQ-5D-5L)

- We would like to know how good or bad your health is **TODAY**.
- This scale is numbered from 0 to 100.
- 100 means the best health you can imagine.
  0 means the worst health you can imagine.
- Mark an X on the scale to indicate how your health is **TODAY**
- Now, please write the number you marked on the scale in the below.

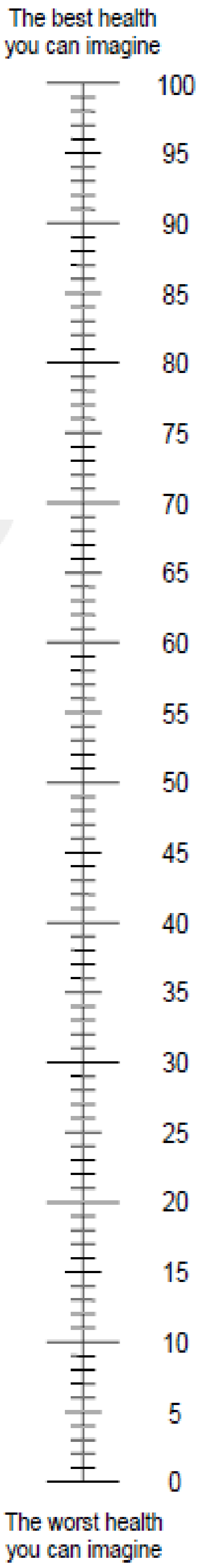

YOUR HEALTH TODAY = ☐

UK (English) © 2009 EuroQol Group EQ-5D™ is a trade mark of the EuroQol Group
