# Supplementary material for: Food Intake Changes and Their Impact on Quality of Life in Spanish Citizens with and without COVID-19 during Lockdown
Source: Healthcare (Basel). 2022 Jul 28;10(8):1414. doi: 10.3390/healthcare10081414 (PMC9408131; doi:10.3390/healthcare10081414)
Supplement: Supplementary file 1 [file healthcare-10-01414-s001.zip › Supplementary material Table S3.pdf]

Table S3. Analysis of differences between independent samples in the AEBQ.

	AEBQ total score during confinement M±SD	*Statics*	*p*[a]
**Sex**			
before pandemic			
Men	61,94±7,518	432,000	**0,033**
Women	66,17±6,623		
during pandemic		502,500	0,409
Men	61,05±12,853		
Women	60,56±5,371		
**COVID-19**			
before pandemic			
People who were not diagnosed	65,41±7,067	852,000	0,914
People who were diagnosed	64,93±7,002		
during pandemic			
No	62,46±13,469	415,000	**0,001**
Yes	58,00±5,831		
**Range of age**			
before pandemic		681,500	0,075
18-29 years	66,28±7,395		
30-68 years	64,00±6,459		
during pandemic		537,000	**0,012**
18-29 years	62,88±11,825		
30-68 years	58,49±10,936		
**Place of residence**			
before pandemic			
North	64,33±6,429	3,279	0,194
Center	64,64±7,442		
South	70,50±2,121		
during pandemic			
North	63,00±8,888	1,918	0,383
Center	57,55±5,475		
South	59,00±0,000		
**Physical activity**			
Before pandemic			
4 or more hours per week	64,74±6,488	1,319	0,725
2-3 hours per week	66,77±66,704		
1 hour per week	64,10±6,774		
No physical exercise	65,31±6,411		

during confinement		7,225	0,065
Less than usual	61,80±9,279		
As before	64,20±7,569		
More than before	61,93±12,795		
No physical activity	54,23±14,613		

Adult Eating Behaviour Questionnaire, AEBQ.
